# Negative Emotion Regulation in Patients with Posttraumatic Stress Disorder

**DOI:** 10.1371/journal.pone.0081957

**Published:** 2013-12-13

**Authors:** Kunlin Xiong, Ye Zhang, Mingguo Qiu, Jingna Zhang, Linqiong Sang, Li Wang, Bing Xie, Jian Wang, Min Li

**Affiliations:** 1 Department of Radiology, Daping Hospital, Third Military Medical University, Chongqing, China; 2 Department of Medical Image, College of Biomedical Engineering, Third Military Medical University, Chongqing, China; 3 Department of Radiology, Southwest Hospital, Third Military Medical University, Chongqing, China; 4 Department of Psychology, Third Military Medical University, Chongqing, China; Banner Alzheimer’s Institute, United States of America

## Abstract

**Objective:**

To explore the neural mechanisms of negative emotion regulation in patients with post-traumatic stress disorder (PTSD).

**Methods:**

Twenty PTSD patients and 20 healthy subjects were recruited. Event-related functional magnetic resonance imaging (fMRI) was used to investigate the modification of emotional responses to negative stimuli. Participants were required to regulate their emotional reactions according to the auditory regulation instructions via headphones, to maintain, enhance or diminish responses to negative stimuli during fMRI scans.

**Results:**

The PTSD group showed poorer modification performance than the control group when diminishing responses to negative stimuli. On fMRI, the PTSD group showed decreased activation in the inferior frontal cortex, inferior parietal lobule, insula and putamen, and increased activation in posterior cingulate cortex and amygdala during up-regulation of negative emotion. Similar decreased activation regions were found during down-regulation of negative emotion, but no increased activation was found.

**Conclusion:**

Trauma exposure might impair the ability to down-regulate negative emotion. The present findings will improve our understanding of the neural mechanisms of emotion regulation underlying PTSD.

## Introduction

Exposure to a traumatic event, such as combat, violent crime, childhood abuse or a motor vehicle accident can result in post-traumatic stress disorder (PTSD), which is characterized by unique symptoms such as the recurrent, involuntary recollection of the trauma in the form of intrusive thoughts, nightmares, and vivid sensory memories [1]. Motor vehicle accidents are the leading cause of PTSD in the general population [2]–[4], which has been studied since the 1980s [5].

Previous fMRI studies have indicated that PTSD is involved in defective emotion regulation [6]–[7]. Emotion regulation is the ability to respond to the ongoing demands of experience with the range of emotions in a manner that is socially tolerable and sufficiently flexible to permit spontaneous reactions as well as the ability to delay spontaneous reactions as needed [8]. Emotional regulation is a complex process that involves initiating, inhibiting, or modulating one’s state or behavior in a given situation. Collectively, processes that serve an emotion regulation function either up-regulate (i.e. enhance), down-regulate (i.e. diminish), or sustain (i.e. maintain) emotions. Different parts of the brain have been increasingly implicated in emotion regulation processes. The limbic system, such as the amygdala, are important in learned emotional associations that become more automatic over time (i.e. bottom-up emotion generating processes), whereas the frontal lobes as well as the anterior cingulate cortex, have been implicated in the regulation of emotion (i.e. top-down emotion regulation) [6]–[7], [9]–[10]. Under stimuli related to the traumatic context, PTSD patients showed less or even no activation in the medial frontal cortex, but greater activation in the amygdala relative to comparison subjects [6]. Lanius et al. reported that during traumatic memory recall tasks, PTSD patients exhibited less activation in the anterior cingulate gyrus, medial frontal gyrus and parietal areas than the control subjects [7]. In a study of PTSD associated with combat, reduced rostral anterior cingulate blood flow was found in the presence of emotionally relevant stimuli in PTSD patients [9]. Additionally, Kim et al. found PTSD patients showed a decreased rostral anterior cingulate function, and the level of decrease in the rostral anterior cingulate activity was negatively correlated with PTSD symptom severity, providing evidence that the rostral anterior cingulate function was impaired in PTSD patients during response–conflict situations involving emotional stimuli [10]. Etkin and Wager found only patients with PTSD showed hypoactivation in the dorsal and rostral anterior cingulate cortices and the ventromedial prefrontal cortex, structures linked to the experience and regulation of emotion, suggesting a mechanism for the emotional dysregulation symptoms in PTSD that extend beyond an exaggerated fear response [11]. These studies only investigated emotion regulation in passive responses to negative stimuli. However, little is known about the neuromechanism underlying voluntary emotion regulation in PTSD patients.

Voluntary emotion regulation in healthy individuals has gained more attention in recent years. Some studies have indicated that healthy individuals can voluntarily regulate their negative emotional responses and successfully suppress negative effects that can lead to decreased physiological activity and more intense negative effects [12]–[15]. In healthy individuals, regions of the prefrontal cortex (PFC), including the orbital frontal cortex and anterior cingulate cortex (ACC), were recruited during down-regulation of negative emotion [12], [15]. The similar regions were also recruited during up-regulation of negative emotion [15]–[16]. Functional neuroimaging studies about voluntary emotion regulation in healthy subjects have found that regions of the PFC are activated, and the amygdala activation is modulated up or down depending on the regulatory goal [14]–[15], [17]. In addition, our previous study also showed that the PFC and basal nuclei were activated during regulation of negative emotion in healthy volunteers, and deliberate down-regulation of emotional responses required participation of more PFC regions [18]. The ability to successfully regulate emotion has been linked to enhanced control of emotion [19], and can be postulated as a protective strategy facing negative stimuli [20]–[21], deficient emotion regulation is thought to be a core mechanism of mood and anxiety disorders [22].

Investigating the voluntary emotion regulation in PTSD patients will not only contribute to understanding the pathomechanism of PTSD, but also aid in the diagnosis and treatment for PTSD. Up to now, there are only two prior studies exploring voluntary emotion regulation in PTSD patients. The first study examined deliberate control of trauma-related mental images and found that among participants with PTSD after missile attacks during the Gulf War, a higher level of image control was related with fewer re-experiencing symptoms [23]. New et al. also investigated the deliberate emotion regulation in women with and without PTSD after sexual trauma, and showed that trauma-exposed individuals (PTSD or non-PTSD) showed worse down-regulation of emotional responses to negative pictures than healthy controls, as measured by subjective rating and PFC activation [24]. The authors suggested that successful down-regulation of emotional responses to negative stimuli appears to be impaired by trauma exposure. Although the two studies help to enrich our understanding of PTSD, the neural bases of the voluntary emotion regulation in PTSD are still unclear.

In the present study, we employed fMRI to investigate the neural mechanisms for the deliberate modification of emotional responses to negative stimuli in PTSD patients. For this purpose, we examined activation of brain regions after instruction to decrease or increase responses to negative emotional stimuli in individuals with PTSD after motor vehicle accidents. Based on the clinical manifestations of PTSD and previous findings, we hypothesized that: (1) The subjective rating to negative stimuli would be significantly different between PTSD and healthy subjects; (2) PTSD patients would show weaker activation in the prefrontal and parietal cortex than healthy subjects during both up- and down-regulation of negative emotion.

## Methods

### Subjects

The subjects in this study had also participated in our previous study [25]. Twenty PTSD patients (range, 18–40 years; mean, 32.92 years) who had been involved in motor vehicle accidents were recruited from Southwest Hospital at Third Military Medical University. Diagnosis of PTSD was established with the Clinician-Administered PTSD Scale for DSM-IV (CAPS-DX) [1]. The CAPS is a structured interview providing a categorical diagnosis, as well as a measure of the severity of PTSD symptoms as defined by DSM-IV. It contains 34 questions, 17 of which measure symptom frequency and 17 measure symptom intensity. The scoring rule is to count a symptom as present if it has a frequency of 1 or more and an intensity of 2 or more. A PTSD diagnosis is made if there is at least 1 “B” symptom, 3 “C” symptoms, and 2 “D” symptoms as well as meeting the other diagnostic criteria. Severity scores can also be calculated by summing the frequency and intensity ratings for each symptom [1]. These interviews conducted in Chinese, the Chinese Version of CAPS is translated from English versions of Blake et al. [1], [26]–[27].

Patients had no history of Axis I psychiatric diagnoses other than depression on the Structured Clinical Interview for DSM-IV (SCID) Axis I Disorders [28], whereas controls were free from Axis I diagnoses on the SCID. The SCID contains a PTSD-specific module with 19 items. Twenty healthy controls (age, 20–38 years; mean, 31.53 years) individually matched by age, gender and years of education were consecutively recruited from the community. Inclusion criteria for all the subjects were right-handedness and an IQ >80, as assessed with the Wechsler Adult Intelligence Scale (WAIS). Exclusion criteria for both groups were contraindications for MRI and other neuropsychiatric disorders, such as schizophrenia, mental retardation, epilepsy, and head injury (i.e., abnormalities on CT or MRI, neurological abnormality during Emergency Department evaluation, posttraumatic amnesia, loss of consciousness for more than 5 min during the accident, or Glasgow Coma Score less than 14). All PTSD patients were diagnosed for the first time during the investigation and had never taken psychotropic medication.

### Ethics Statement

This research was conducted in accordance with international ethical guidelines for biomedical research involving human subjects, and approved by the ethics committee of Third Military Medical University. All participants gave written informed consent after receiving a complete description of the study.

### Experimental Stimuli and Design

**Figure 1:**
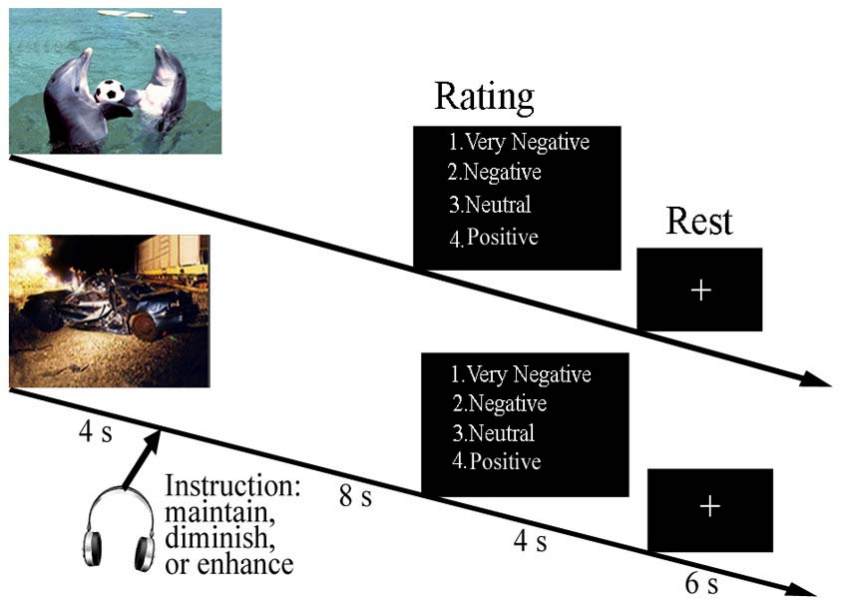
Task design for emotion modification. Examples of two trials are shown, one with the neutral picture and the other with the negative picture. Here, the representative images are similar to the IAPS image used in the task. There are three instructions, including “diminish”, “maintain”, and “enhance”. The bottom picture is an example of a negative image, to depict trauma-related negative stimuli. For neutral pictures, subjects were asked only to “maintain” their responses.

During fMRI scans, participants were required to regulate their emotional reactions to neutral and negative pictures according to the auditory regulation instructions (maintain, enhance or diminish) via headphones, as used by New et al. [24]. During each trial, subjects viewed 3 sets of 5 neutral and 15 negative pictures. For the neutral pictures, subjects received only the maintain instruction. For the negative pictures, they received one of the three regulation instructions with five pictures for each condition, e.g. to “diminish”, “enhance”, or “maintain” their responses. Negative pictures were matched for valence and arousal across regulation instruction conditions. To diminish their response, subjects were instructed to decrease the intensity of the negative effect of images by imagining a less negative outcome for the circumstances depicted in the picture. Conversely, to enhance their response, subjects were instructed to imagine a more negative outcome. In the maintain condition, participants were instructed to maintain their responses. During each trial, pictures were randomly assigned to regulation conditions on a subject-by-subject basis. Before the intertrial interval, subjects rated their emotional experience on a Likert-type scale by indicating how negative or positive they found the picture (1: very negative; 2: negative; 3: neutral; 4: positive; 5: very positive). In this study, the scale ranged from 1 to 4, since only neutral and negative pictures were included. Each trial consisted of 1-s fixation, a 12-s picture period, a regulation instruction delivered 4 s after picture onset, a 4-s screen displaying a scale for subjective rating, and a 6-s rest (Figure 1; fixation period not shown). Trials were presented in 3 runs of 20 pictures, with the instructions presented in a pseudorandom order to maximize the fMRI model estimation efficiency. Prior to scanning, participants were trained to perform the emotion regulation.

The negative and neutral pictures were selected from the International Affective Picture System (IAPS) [29]. The arousal and valence scales, respectively, were as follows: 6.23±0.26/2.17±0.34 for negative pictures, and 4.18±0.72/5.12±1.04 for neutral pictures.

### Image Acquisition

All experiments were performed on a 3.0 T Siemens MRI scanner (Trio, Siemens Medical Erlangen, Germany). Foam padding was used to minimize head motion for all subjects. The fMRI data were acquired using the following parameters: TR/TE/FA 2000 ms/30 ms/90°, 36 transverse slices, thickness of 3.0 mm, FOV of 220×220 mm. T1-weighted images in the sagittal plane of all subjects were acquired using a 3D MPRAGE sequence with TR/1900 ms, TE/2.34 ms, flip angle/7°, FOV/256×256, and slice thickness/1 mm.

### Image Processing and Analysis

Image preprocessing and statistical analysis were performed using SPM8 software (http://fil.ion.ucl.ac.uk.spm/). For each subject, standard steps for preprocessing the EPI images were conducted, including correction for slice timing and head motion, registration to a high-resolution anatomical image, spatial normalization, and smoothing using a Gaussian kernel of 8-mm full-width at half maximum. A temporal high-pass filter with a period cutoff of 128 s was also applied. This was followed by the whole-brain voxel-based general linear model at the single-subject level to estimate signal change associated with the conditions of interest (e.g., negative pictures during the diminish condition) with regard to the baseline condition, using six motion parameters as covariates of no interest. Specifically, one regressor was used to model the fixation period before the image using a boxcar function of 1-s duration; two regressors were used to model the initial picture periods for negative and neutral images using boxcar functions of 4 s; three regressors were used to model the three different sound instructions using 1-s boxcar functions; four regressors using 7-s boxcar functions were used to model the regulation periods for the negative-diminish, negative-enhance, negative-maintain, and neutral-maintain condition. For individual analyses, the fMRI signal was selectively averaged in each subject as a function of emotion regulation (i.e., negative-diminish, negative-enhance, negative-maintain, neutral-maintain).

The outputs of individual analyses were used as inputs for second-level random-effects group analyses, and two-sample t statistics for the contrast of interest (negative-diminish, negative-enhance, negative-maintain, neutral-maintain) were calculated for cross-group comparison. An intensity threshold of p<0.01 and an extent threshold of 20 contiguous voxels were used for correction during multiple voxel comparisons. The t-map was set at a corrected threshold of p<0.05 (combined height threshold p<0.01 and a minimum cluster size of 20 voxels), using the AlphaSim program in the REST software (http://www.restfmri.net/forum/REST_V1.8), which applied Monte Carlo simulation to calculating the probability of false positive detection by considering both the individual voxel probability thresholding and cluster size.

### Statistical Analysis

The subjective rating scores of each subject were calculated by Excel 2007, and analyzed by SPSS for Windows v17.0 (SPSS Inc., Chicago, Illinois). All results are quoted as 2-sided *p* values *p*<0.05 was considered statistically significant.

## Results

### Subject Characteristics

PTSD patients and the controls were matched with respect to age, gender, and years of education (*p*>0.05), and there were no significant differences in IQ between the two groups (*p*>0.05). Patients with PTSD had significantly greater CAPS scores (*p*<0.05) (Table 1). According to the SCID, 3 subjects in the PTSD group met DSM-IV diagnostic criteria for depressive disorder. Among our control subjects, the SCID did not reveal any psychiatric disorders.

**Table 1:** Demographic and clinical characteristics of PTSD patients and the controls.

Variable	PTSD (n = 20)	Controls (n = 20)	*p* value*
Mean age in years (SD)	32.92 (8.48)	31.53 (7.43)	0.45
Gender	Male (13), Female (7)	Male (14), Female (6)	0.74
Mean education in years (SD)	11.20 (3.80)	13.00 (2.20)	0.37
IQ (SD)	98.20 (5.50)	103.20 (6.30)	0.24
CAPS total score, mean (SD)	52.33 (9.44)	8.26 (9.31)	0

–136). CAPS, Clinician-Administered PTSD Scale (range, 0

*p* Values are calculated by χ^2^ statistics for categorical measures and two-tailed t statistics for continuous measures.

### Subjective Rating

The subjective rating scores of the control group to negative pictures were 1.75±0.41 (maintain), 2.50±0.61 (diminish) and 1.35±0.51 (enhance), the rating scores of the PTSD group were 1.71±0.57 (maintain), 1.98±0.42 (diminish) and 1.30±0.49 (enhance). Within group analysis showed significant differences between negative-enhance and negative-maintain (*p*<0.05), and between negative-diminish and negative-maintain in the healthy controls (*p*<0.05). In the PTSD group, there was a significant difference between negative-enhance and negative-maintain (*p*<0.05), but no difference between negative-diminish and negative-maintain (*p*>0.05). Group comparison analysis showed no differences in subjective ratings between the PTSD and the control group in both negative-enhance and negative-maintain (*p*>0.05). While for the negative-diminish, there was a significant difference between the two groups (*p*<0.05). These findings suggested that the controls could successfully down-regulate or up-regulate negative emotion, the PTSD patients could up-regulate their reactions to negative stimuli as successfully as the controls, but could not down-regulate reactions to negative stimuli.

### fMRI Results

**Figure 2:**
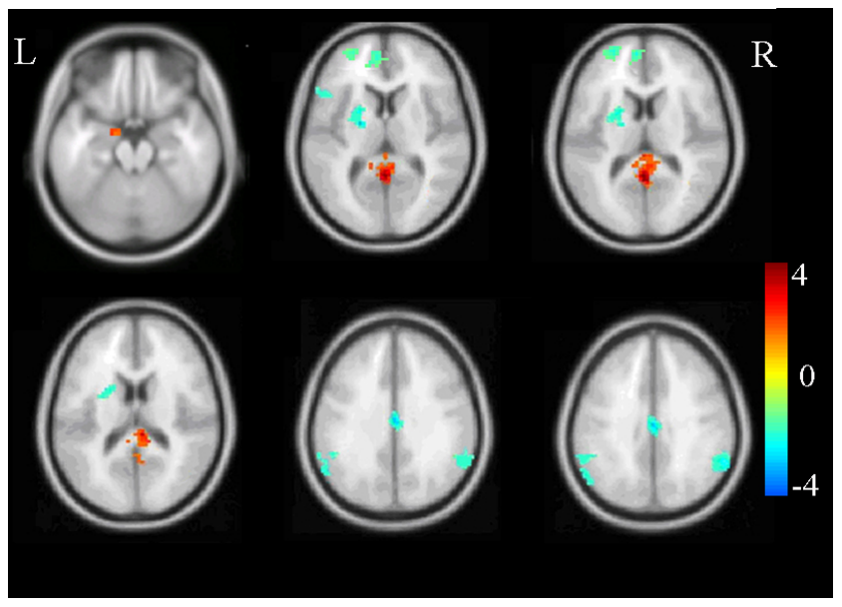
Group comparison in the negative-enhance condition. A warm color indicates increased activation, and cold color indicates decreased activation. The color bar indicates T-score. L:left, R:right.

**Figure 3:**
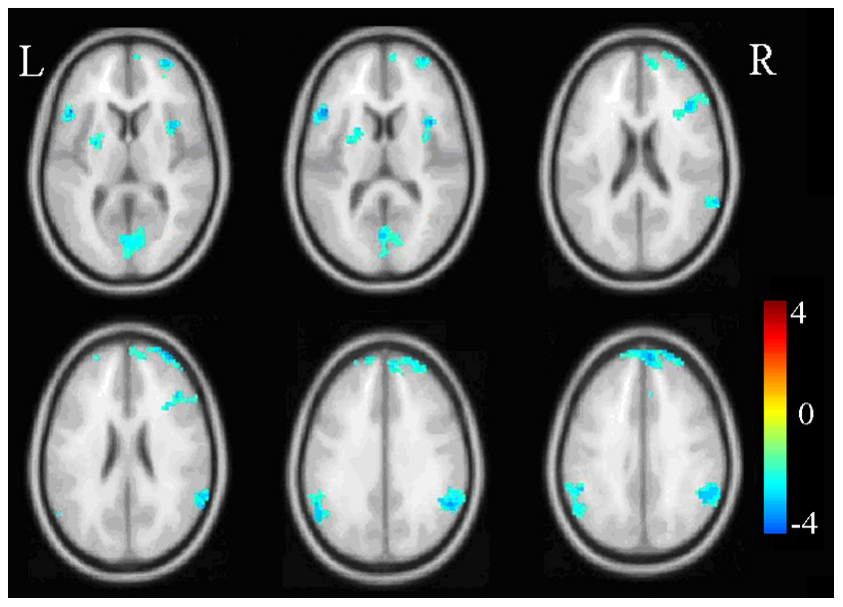
Group comparison in the negative-diminish condition. The cold color indicates decreased activation. The color bar indicates T-score. L:left, R:right.

We mainly examined group differences in activation of brain regions in the different emotion regulation conditions. For both negative-maintain and neutral-maintain, there were no group differences in activation. In the negative-enhance condition, PTSD patients showed increased activation in the amygdala and posterior cingulate cortex, and decreased activation in the anterior cingulate cortex, middle cingulate cortex, left inferior frontal cortex, left putamen and bilateral inferior parietal lobule (p<0.05) (Fig. 2, Table 2). In the negative-diminish condition, no increased activation was found in the PTSD patients compared with the healthy controls, while regions with decreased activation were found in the inferior frontal cortex, left putamen, bilateral inferior parietal lobule, and insula (Fig. 3, Table 2).

**Table 2:** Significant clusters identified in PTSD patients compared with controls.

		Anatomic definition	Brodmann area	Voxels	P value	MNI coordinates		
						X	Y	Z
Up-regulate	ptsd>control	posterior cigulate cortex	31	97	0.01	2	−42	10
		amygdala	-	12	0.04	−21	−2	11
	ptsd<control	middle frontal lobe	10	85	0.03	−33	52	15
		anterior cigulate cortex	32	65	0.04	−2	39	6
		inferior parietal lobe(right)	40	69	0.02	48	−45	42
		inferior parietal lobe(left)	40	91	0.04	−47	−43	41
		middle cigulate cortex	23	68	0.02	−1	−18	38
		Putamen	–	57	0.04	−24	3	12
		inferior frontal lobe	45	50	0.03	−50	25	15
Down-regulate	ptsd<control	inferior parietal lobe(right)	40	76	0.04	47	−52	41
		inferior parietal lobe(left)	40	95	0.03	−51	−50	39
		inferior frontal lobe	45	60	0.01	51	27	18
		middle frontal lobe	9	105	0.03	31	57	13
		superior frontal lobe	10	117	0.03	6	55	36
		putamen	–	57	0.02	−26	4	22
		insula	13	43	0.03	36	9	12
		cuneus	18	88	0.04	2	−74	12

## Discussion

The present study investigated the neural mechanisms for the voluntary regulation of emotional responses to negative stimuli in PTSD using fMRI. Consistent with previous findings, the healthy controls appeared to be more successful at diminishing emotional responses to negative stimuli than PTSD, as measured by subjective reactions and region activation in the prefrontal cortex. Moreover, in both up- and down-regulation of negative emotion conditions, PTSD patients showed less activation in the prefrontal and parietal cortex than healthy subjects. These findings verified our hypotheses.

The ability to regulate emotional responses to aversive events is important for mental and physical health as well as social interaction [30]. Successful down-regulation of emotional reactions to aversive events can help individuals minimize negative, distressing emotions [19], [31]. Conversely, disruptions in normal emotion regulation are associated with the genesis and maintenance of depression and anxiety, both of which may involve a chronic inability to suppress negative emotion [12], [22]. PTSD is a severe anxiety disorder that can develop after exposure to any event that causes psychological trauma [32]. When exposed to reminders of traumatic events, PTSD patients can easily become depressed, negative, irritable, and fail to get over negative emotion [33]. Indeed, we found that PTSD patients showed less effective attenuation of responses to trauma-related negative stimuli as measured by subjective reactions and activation in the prefrontal cortex compared with the healthy controls, suggesting an impaired ability to down-regulate negative emotion in PTSD patients. The findings are consistent with previous studies [11], [24], supporting that trauma exposure might impair the ability to down-regulate negative emotion. Similar with the study by New et al., we found no significant group differences in amygdala activation during the down-regulation condition, indicating that the PTSD patients and the healthy control groups might employ different brain regions in regulatory strategies [24].

To further examine the neural mechanism of voluntary emotion regulation using fMRI, we found that under both up- and down-regulation conditions, PTSD patients showed weaker activation in the prefrontal and parietal cortex compared with the healthy controls, verifying our second hypothesis. Ochsner et al. investigated the neural bases of emotion regulation in the healthy subjects and found that both up- and down-regulation of emotion was associated with increased activity in the prefrontal and anterior cingulate regions [15]. In addition, our previous results showed that more regions in the prefrontal and parietal cortex related to cognitive control were activated during up- and down-regulation in the healthy subjects [19]. Several studies have consistently found that the voluntary enhancement of responses to negative stimuli can decrease the intensity of negative emotion [34]–[36], indicating that the ability to focus on negative emotions helps to extinguish negative emotional responses [24]. The present findings of reduced activation in the prefrontal and parietal regions during up- and down-regulation conditions suggest a deficit in voluntary modification of negative emotion in PTSD patients, since alexithymia is a risk factor for PTSD [35]–[36]. Furthermore, our previous study analyzing cortical thickness in the same participants found significantly decreased cortical thickness in the left medial prefrontal cortex and anterior cingulated cortex in PTSD patients, suggesting deficits in working memory of PTSD patients [25]. These might be the structural bases in the brain accounting for the impaired ability to voluntarily regulate negative emotion in PTSD patients.

Additionally, greater activation was found in the amygdala and posterior cingulate cortex (PCC) after the “enhance” instruction to negative stimuli in PTSD patients than the healthy controls. For the healthy subjects, cognitive up-regulation of negative emotion is associated with greater amygdala activation [15]. Here, the greater activation of the amygdala and PCC in PTSD patients might be due to the weaker activation in the prefrontal and parietal regions. The prefrontal regions normally inhibit the amygdala. Our findings suggest that the prefrontal and parietal cortex related to cognitive control could not effectively inhibit the activation of the amygdala and PCC, and results in much slower extinction of aversive responses in PTSD patients [37]. The greater activation of the PCC in PTSD patients also supports the prominent role for the PCC in pain and episodic memory retrieval [38]. The episodic memory is a key factor in the aggravation and maintenance of PTSD symptoms [39]. The negative pictures used in this study were related to motor vehicle accidents, which might remind the PTSD patients of their painful experience. Therefore, we found substantial activation of the PCC in PTSD patients after enhancing emotion responses to negative pictures.

It is worth noting that individuals differ in their use of emotion down regulation strategies. There are two commonly used strategies for down-regulating emotion [40]. The first is cognitive reappraisal, which is a type of cognitive change, and thus antecedent focused. Reappraisal is defined as construing a potentially emotion-eliciting situation in nonemotional terms, and participants are asked to feel less emotion. The second is expressive suppression, which is a type of response modulation, and thus response focused. Suppression is defined as inhibiting ongoing emotion-expressive behavior, and participants were asked to hide their emotional reactions. In our experiment, when down-regulating negative emotion (diminish their response), subjects were instructed to decrease the intensity of the negative effect of images by imagining a less negative outcome for the circumstances depicted in the picture. This emotion regulation strategy in our study is not “suppression”, but more close to “reappraisal”. Experimental and individual-difference studies find reappraisal is often more effective than suppression. Reappraisal decreases emotion experience and behavioral expression, and has no impact on memory [41]. By contrast, suppression decreases behavioral expression, but fails to decrease emotion experience, and actually impairs memory [41]. Appleton et al. also reported that emotional suppression was associated with elevated inflammation, shown this maybe a maladaptive emotion regulation strategy in some people [42]. Up to now, it is still not clear what role the different strategy for down regulation negative emotion affects PTSD patients; this would be an important area for future research on emotion regulation processes and psychological treatment on PTSD patients.

Our study has a number of limitations. First, a small number of participants were included in this study, and the psychometric properties of PTSD patients were not documented, which might limit the generalizability of the findings. Future studies should employ a larger sample, and collect the psychometric properties of PTSD patients. Second, we only included healthy participants in the control group to investigate the neural mechanism of voluntary emotion regulation to negative stimuli in PTSD patients. It may be that individuals with traumatic exposure and no PTSD also experience increases in emotional reactivity/intensity following trauma cue exposure. Comparative analysis including non-PTSD patients undergoing traffic trauma rehabilitation would help us better understand the pathomechanism of PTSD. Lastly, it is possible that these findings were related to difficulties regulating trauma-related emotion (vs. negative emotions more generally) because some of the pictures were accident-related in our experiment. In the future, it is worth to study the difference between the trauma-related emotion regulating and negative emotion regulating in PTSD patients.

In conclusion, the present study investigated the neural mechanisms for the voluntary regulation of emotional responses to negative stimuli in patients with PTSD after motor vehicle accidents. Our findings supported that trauma exposure might impair the ability to downregulate negative emotion. Furthermore, the results of decreased activation in the prefrontal and parietal cortex in PTSD patients during voluntary regulation of negative emotion extend the research on the neural mechanism of passive responses to negative stimuli. Our results are consistent with previous studies on emotion regulation in PTSD with different subtypes, suggesting that PTSD with different traumas might share a similarly impaired neural mechanism of emotion regulation. The present findings will improve our understanding of the neural mechanisms of emotion regulation underlying PTSD.
